# Validation of Image Qualities of a Novel Four-Mice Bed PET System as an Oncological and Neurological Analysis Tool

**DOI:** 10.3390/jimaging7030043

**Published:** 2021-02-26

**Authors:** Kyung Jun Kang, Se Jong Oh, Kyung Rok Nam, Heesu Ahn, Ji-Ae Park, Kyo Chul Lee, Jae Yong Choi

**Affiliations:** 1 Division of Applied RI, Korea Institute of Radiological and Medical Sciences, Seoul 01812, Korea; kangkj1@kirams.re.kr (K.J.K.); osj5353@kirams.re.kr (S.J.O.); krnam@kirams.re.kr (K.R.N.); heesu625@kirams.re.kr (H.A.); jpark@kirams.re.kr (J.-A.P.); kyochul@kirams.re.kr (K.C.L.); 2 Radiological and Medico-Oncological Sciences, University of science and technology (UST), Seoul 01812, Korea

**Keywords:** positron emission tomography, multi-bed system, oncology, neurology

## Abstract

**Background**: Micro-positron emission tomography (micro-PET), a small-animal dedicated PET system, is used in biomedical studies and has the quantitative imaging capabilities of radiotracers. A single-bed system, commonly used in micro-PET, is laborious to use in large-scale studies. Here, we evaluated the image qualities of a multi-bed system. **Methods**: Phantom imaging studies were performed to assess the recovery coefficients (RCs), uniformity, and spill-over ratios (SORs) in water- and air-filled chambers. ^18^F-FDG and ^18^F-FPEB PET images of xenograft and normal mice from the multi-bed and single-bed systems were compared. **Results**: For small diameters (< 3 mm), the RC values between the two systems differed significantly. However, for large diameters (> 4 mm), there were no differences in RC values between the two systems. Uniformity and SORs of both systems were within the tolerance limit of 15%. In the oncological study, the estimation of ^18^F-FDG uptake in the tumor was significantly lower in the multi-bed system than that in the single-bed system. However, ^18^F-FDG PET in xenograft mice with tumor size > 4 mm revealed the variation between subjects within the multi-bed system group to be less than 12%. In the neurological study, SUV for the multi-bed group was 25–26% lower than that for the single-bed group; however, inter-object variations within the multi-bed system were below 7%. **Conclusions**: Although the multi-bed system showed lower estimation of radiotracer uptake than that of the single-bed system, the inter-subject variations were within acceptable limits. Our results indicate that the multi-bed system can be used in oncological and neurological studies.

## 1. Introduction

Molecular imaging is used for the diagnosis of oncological, neurological, and cardiovascular diseases [1,2,3,4]. Positron emission tomography (PET) is a molecular imaging technique that enables quantitative measurements of biochemical parameters, such as metabolic changes and concentrations of neurotransmitters [5,6,7]. The demand for preclinical research is increasing worldwide, as it can predict clinical trials and advance our understanding of the pathophysiological mechanisms underlying a specific disease [8].

For this purpose, researchers use micro-PET (µPET), which is a small-animal dedicated system. µPET vendors provide a single bed, thereby allowing imaging of only a single animal at a time. Large-scale research involving many objects thus requires tremendous time and use of radioactivity [9]. Sometimes, house-made multibed systems are used, but these systems are difficult to use with other µPET imaging systems. Recently, Mediso^TM^ (nanoScan^®^, Mediso Medical Imaging Systems, Budapest, Hungary) developed a multi-bed system dedicated to PEC/CT scanners, and its initial validity for research has been investigated. Hannah et al. tested a four-bed mouse system using 2-deoxy-2-(^18^F)fluoro-D-glucose (^18^F-FDG) in phantom and normal mice and reported a quantitative accuracy similar to that of a single-bed [10]. To date, however, few studies have focused on the validity of oncological and neurological PET imaging of the four-mice bed system. 

This study aimed to evaluate the image qualities of oncological and neurological PET imaging using a novel four-mice bed system.

## 2. Materials and Methods

### 2.1. Mini Image Quality Phantom

We used a cylindrical image quality phantom developed by Mediso^TM^ (Budapest, Hungary), specifically designed for use in four-mice beds. The phantom mimics the shape of the mouse body and is built of a flexible glass material. It consists of three parts: A large uniform compartment, five rods (1, 2, 3, 4, and 5 mm), and a non-radioactive region containing water and air-filled chambers. The diameter and volume of the phantom were 20 and 10 mL, respectively. The diameter of the spill-over-ratio chambers was 5.5 mm.

### 2.2. Animals

All experimental procedures were approved by the Institutional Animal Care and Use Committee of the Korea Institute of Radiological & Medical Sciences (IACUC permit number: KIRAMS 2020–0033, 2020-05-22) and conducted in accordance with the National Institutes of Health Guidelines for the Care and Use of Laboratory Animals. Female BALB/c nude mice (age, 6 weeks; weight, 18–20 g; n = 12 for tumor study; n = 8 for brain PET study) were purchased from Central Lab. Animal, Inc. (Seoul, Korea). For the tumor study, a single-cell suspension (4 × 10^6^ cells) was subcutaneously injected into the flanks of 7-week-old mice. Animals were housed in the Institutional Biological Rodent Unit on a 12-h light/dark cycle at room temperature with free access to standard laboratory chow and tap water. They were acclimatized to laboratory conditions for 7 days prior to the experiments.

### 2.3. Radiosynthesis of PET Tracers

We used two types of radiotracers. ^18^F-FDG is a specific biomarker of glucose metabolism. 3-[^18^F]fluoro-5-(2-pyridinylethynyl)benzonitrile (^18^F-FPEB) is a specific radiotracer for metabotropic glutamate receptor subtype 5 (mGluR5). Both radiotracers were prepared by nucleophilic substitution of ^18^F into its precursors (i.e., mannose triflate for ^18^F-FDG and acetonitrile or dimethyl sulfoxide (DMSO) for nitro-FPEB). After radiosynthesis, we performed quality control examinations. The formulated radiotracers were colorless solutions, showing pH of 7 and 511 keV in a multi-channel analyzer. We found acetonitrile < 400 ppm and DMSO < 5000 ppm as residual solvents. The concentration of endotoxins was below 1.01 EU/mL and both formulated radiotracers passed the sterility test. All radiotracers were prepared according to a previously described procedure [11,12]. The radiochemical purity of the radiotracers was >99%.

### 2.4. PET Imaging

#### 2.4.1. Phantom Study

**Figure 1:**
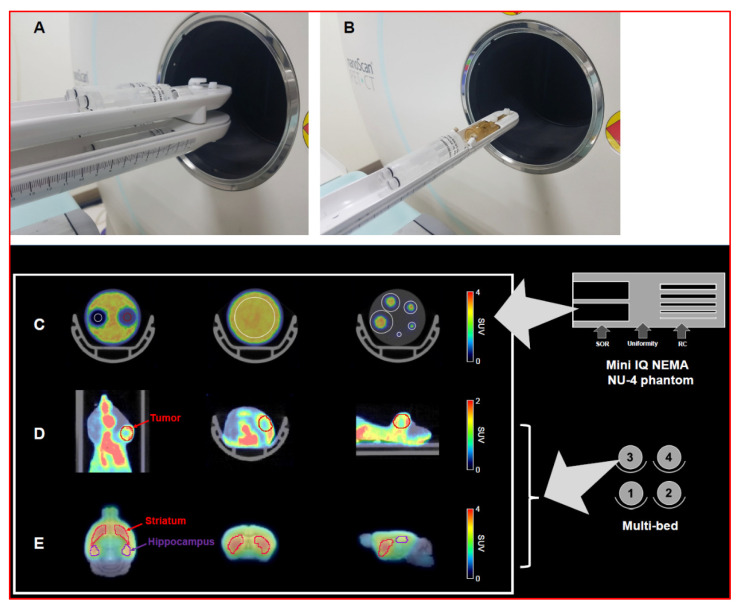
Design of the multi-bed (**A**) and single-bed PET imaging system (**B**). Definition of the VOIs in the mini IQ NEMA Nu-4 image quality phantom (**C**) each volume of interest (VOI)was drawn to calculate following factors; spill-over ratio (left), uniformity (middle), recovery coefficient (right)). The VOIs for the tumor region (**D**) and the brain (striatum and hippocampus, (**E**)) are also represented.

Following the guidelines of NEMA NU-4 2008, four phantoms were filled with 3.7 MBq of ^18^F-FDG in 10 mL of saline and decay-corrected to the start of acquisition. On the first day, four phantoms were imaged in the multi-bed system following the same specifications of the PET manufacturer for single mouse imaging (Figure 1A). After 24 h, a single phantom was imaged using a single-bed system for comparison (Figure 1B). Static PET acquisition was performed using a Mediso nanoScan PET/CT over 20 min, followed by CT (480 projections; 50 kVp tube voltage; 630 µA; 300 ms exposure time; 1:4 binning). Whole-body TeraTomo 3D reconstruction with four iterations and six subsets was performed (1–5 coincidence mode) using an isotropic voxel size of 0.4 mm^3^.

#### 2.4.2. Animal PET Studies

Whole-body PET images of the mice were obtained using the Mediso PET scanner (Mediso Medical Imaging Systems, Budapest, Hungary). In all the experiments, four animals were scanned in the multi-bed system, and then, single PET images were acquired using the single-bed system 2 days later. For the oncological study, prior to imaging, all mice fasted overnight. The mice were anesthetized with 2.5% isoflurane in oxygen, and ^18^F-FDG (8.8 ± 0.9 MBq/200 µL) was administrated into the tail vein. Based on previous studies, ^18^F-FDG images were acquired after 40–60 min [13]. For the brain study, an ^18^F-FPEB PET tracer (8.7 ± 0.7 MBq/200 µL) was prepared. ^18^F-FPEB PET images were acquired 30–50 min after injection because the radiotracer initially shows specific-to-nonspecific binding values that reach a plateau after 30 min. The scanner has a peak absolute system sensitivity of >9% in the 400–600 keV energy window, an axial field of view of 28 cm, a transaxial field of view of 35–120 mm, and a transaxial resolution of 0.7 mm at 1 cm off-center. Images were reconstructed using a three-dimensional ordered subset expectation maximization (3D-OSEM) algorithm with four iterations. For attenuation correction, micro-CT imaging was performed immediately after PET using 50 kVp of X-ray voltage with 0.16 mAs. While imaging, we controlled the internal bed temperature using an air pump (MultiCell™ controlled Large pump, Budapest, Hungary) provided by the manufacturer and monitored the body temperature of the animals in real-time using software (Multicell GUI version 2.4, Budapest, Hungary).

### 2.5. Image Analysis

#### 2.5.1. NEMA NU-4 2008 Standard Process

To assess the scanner performance of the multi-bed system, we performed NEMA NU-4 2008 tests for a small-animal PET system. To evaluate the spatial resolution of PET images using the four-mice bed system, recovery coefficients (RCs) were calculated for rod diameters of 1, 2, 3, 4, and 5 mm and compared with the results of the single-bed system. Circular regions of interest (ROIs) were drawn around each rod, with diameters twice that of the rods (Figure 1C). Next, the RC values were calculated by dividing the maximum activity detected at each rod by the mean phantom concentration. For uniformity calculation, we drew long cylindrical ROIs (75% of the active diameter) in the center of the uniform region of the phantom. Based on the obtained average activity concentration and maximum and minimum values, the percentage standard deviation (%STD_unif_) was calculated. To estimate the accuracy of scatter correction, spill-over ratios in the water (SOR_water_) and air-filled (SOR_air_) cylindrical inserts were estimated as the ratio of the mean activity concentration within the volumes of interest (VOIs) to the mean counts in the uniformity region.

#### 2.5.2. Animal PET

For the tumor PET study, the CT and PET data were co-registered and ellipsoidal VOIs were manually defined for the tumor using PMOD software (version 3.4, PMOD Technologies Ltd, Zurich, Switzerland) (Figure 1D). The maximum standardized uptake value (SUV_max_) within the VOI on the PET image was measured. After the PET imaging, all animals were sacrificed, and the tumor size was measured. Based on phantom imaging results, we classified tumors into small (less than 3 mm) and large (>4 mm) tumor size groups and evaluated the tumor detection capability of the multi-bed system against the single-bed system. The decay-corrected radioactivities were normalized in units of SUV to normalize the differences in injected dose and body weight. The SUV was obtained by dividing the tissue radioactivity concentration by the injected dose and mouse body weight. For mouse brain data analysis, a house-made brain MR template was used [14]. Next, PET images were spatially normalized based on the T2-weighted mouse brain MR template (M. Mirrione), embedded in PMOD software, masked to exclude extra-cerebral signals, and smoothed with a 3D Gaussian filter (full width at half maximum = 1.0 mm). The striatum and hippocampus were selected as VOIs on the MR template (Figure 1E), and the mean SUVs were recorded.

### 2.6. Statistical Analysis

Quantitative results were expressed as mean ± SEM. Student’s *t*-test or one-way ANOVA followed by a Tukey post hoc test were used to determine significant differences between the experimental groups. All statistical analyses were performed using Prism software. 

## 3. Results and Discussion

### 3.1. Phantom Studies

**Figure 2:**
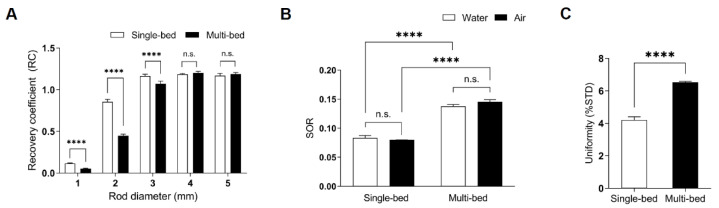
The RC values of 1–5 mm rods (**A**), spill-over ratios (SORs) (**B**), and uniformity values (**C**) of phantoms imaged in the single- and multi-bed systems. Statistical significance was defined as a *p* value less than 0.05 (**** *p* < 0.0001, n.s.= statistically nonsignificant difference).; %STD, percentage standard deviation.

To evaluate the spatial resolution of PET images, the RC values were measured in both single-bed and multi-bed systems. For the 1- and 2-mm rods, RC values of the multi-bed system (0.05 ± 0.01 and 0.45 ± 0.02, respectively) were decreased by 48–58% compared to those of the single-bed system (0.12 ± 0.01, *p* < 0.0001 and 0.86 ± 0.03, *p* < 0.0001 for 1- and 2-mm rods, respectively; Figure 2A). For rods greater than 3 mm, the difference between the systems gradually decreased (single-bed: 1.17 ± 0.02 vs. multi-bed: 1.07 ± 0.03, *p* < 0.0001). For rods above 4 mm, there was no significant difference in the RC values between the two systems (single-bed: 1.19 ± 0.01 and 1.17 ± 0.03 for 4- and 5-mm rods, respectively vs multi-bed: 1.20 ± 0.02, p = 0.085 and 1.19 ± 0.02, *p* = 0.1039 for 4- and 5-mm rods, respectively). Therefore, using multiple beds for sizes above 4 mm is recommended because the RC difference is under 2%. 

To assess scatter correction, SOR values for both water- and air-filled chambers were calculated. The multi-bed system had 65–82% higher values than the single-bed system (SOR_water_ for single-bed: 0.08 ± 0.01 vs. SOR_water_ for multi-bed: 0.14 ± 0.02, *p* < 0.0001; SOR_air_ for single-bed: 0.08 ± 0.01 vs. SOR_air_ for multi-bed: 0.15 ± 0.01, *p* < 0.0001; Figure 2B). However, the values for both systems were within the tolerance limit of 15%, as suggested by the NEMA NU-4 guidelines. These results are consistent with previous findings, indicating that appropriate scatter correction was applied to both systems [10]. 

Regarding uniformity comparison, the %STD value in the multi-bed system was significantly higher than in the single-bed system (%SDT for single-bed: 4.20 ± 0.49 vs. %SDT for multi-bed: 6.53 ± 0.22, *p* < 0.0001; Figure 2C), suggesting that image noise was elevated in the multi-bed system. However, neither system exceeded the tolerance limit of 15%.

Thus, the multi-bed system has a spatial resolution similar to that of the single-bed system for sizes >4 mm and meets the SOR and uniformity value proposed by NEMA.

### 3.2. Animal Studies

#### 3.2.1. Oncological Study

**Figure 3:**
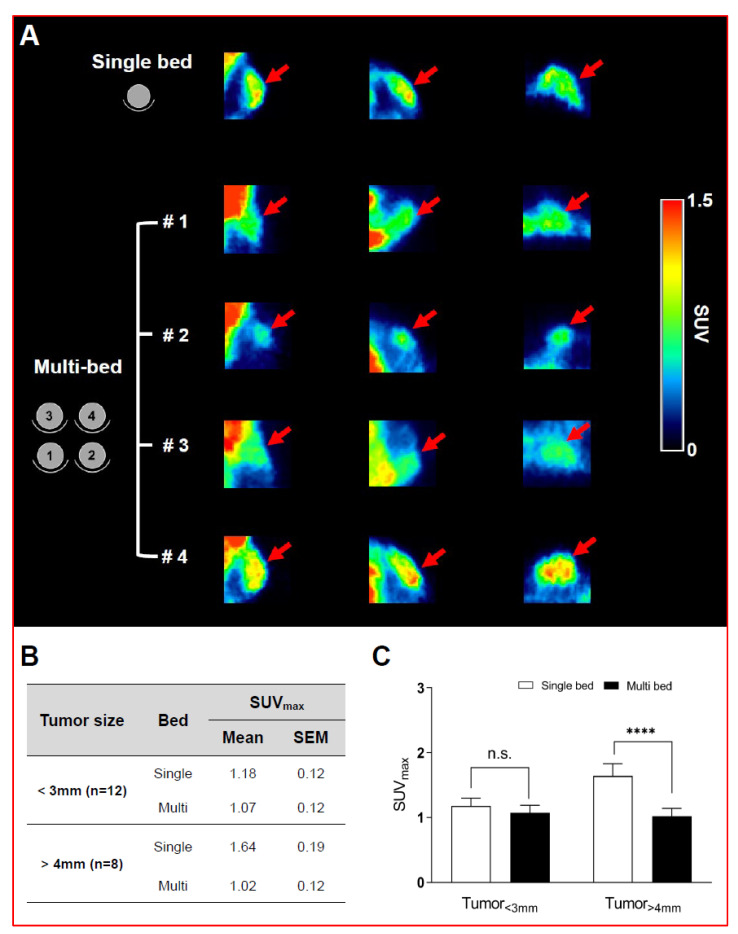
Representative PET/CT images of tumor-bearing mice after ^18^F-FDG injection in single- and multi-bed systems (**A**). The plane was chosen to best reflect the tracer uptake characteristics of each group. Comparison of tumor uptake values in single- and multi-bed systems based on tumor size (**B**,**C**), **** *p* < 0.0001, n.s.= statistically nonsignificant. PET/CT, positron emission tomography/computed tomography; SUV_max,_ maximum standardized uptake value.

A visual comparison of glucose PET (i.e., ^18^F-FDG) in xenograft mice with small tumor size (<3 mm) showed no difference between the single-bed and multi-bed systems (Figure 3A). Indeed, there was no significant difference in the SUV_max_ between the single- and multi-bed systems (single-bed: 1.18 ± 0.12 vs. multi-bed: 1.07 ± 0.12, *p* = 0.1056; Figure 3B,C). These results are consistent with the phantom results, where the reduced spatial resolution was evident in RC examination of the 1- and 2-mm rods. For large tumor-bearing mice (>4 mm), it was possible to discriminate the tumor region in both systems by visual inspection; however, clearly increased radiotracer uptake was shown in the single-bed system compared to that shown in the multi-bed system (Figure 3A). In addition, the SUV_max_ for the multi-bed system was significantly reduced by 38% compared to that for the single-bed system (single-bed: 1.64 ± 0.19 vs. multi-bed: 1.02 ± 0.12, *p* < 0.05, Figure 3B,C). However, the inter-object variation was below 12% for the multi-bed system. Thus, the multi-bed system is recommended when the tumor volume is greater than 4 mm.

#### 3.2.2. Neurological Study

**Figure 4:**
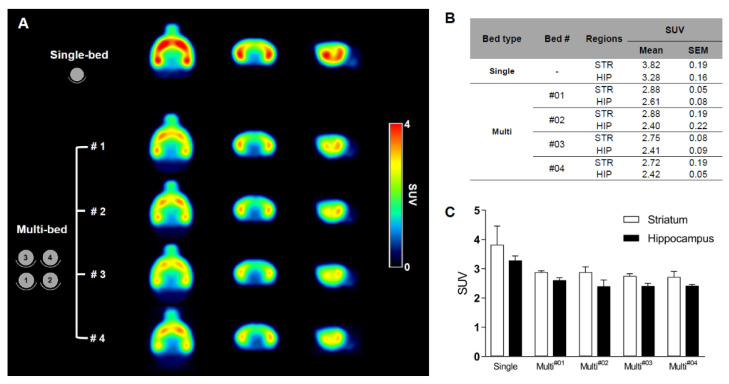
Summed ^18^F-FPEB brain PET images of the single-bed (n = 12) and each bed of the multi-bed system (**A**, n = 3 in each bed). Comparison of regional SUV values of single- and multi-bed systems (**B**,**C**). STR, striatum; HIP, hippocampus; SUV, standardized uptake value.

The mean SUV for the multi-bed group was 25–26% lower than that for the single-bed group (hippocampus: Single-bed, 3.28 ± 0.16 vs. multi-bed, 2.46 ± 0.06, *p* < 0.0001; striatum: Single-bed, 3.82 ± 0.19 vs. multi-bed, 2.81 ± 0.06, *p* < 0.001). The variation in brain PET uptake between subjects was low in both systems (SEM values: 0.05–0.19, Figure 4B); notably, the variation in the multi-bed system was relatively lower than that in the single-bed system (SEM for the hippocampus: Multi-bed, 0.06 and single-bed, 0.16; striatum: Multi-bed, 0.06 and single-bed, 0.19). Within the multi-bed system, the inter-object variation was below 7% (Figure 4B) and there were no significant differences in the target regions across the four different beds (for striatum *p* = 0.4186, for hippocampus *p* = 0.2169).

Thus, the multi-bed system has sufficient stability to resolve in brain PET.

## 4. Conclusions

PET images acquired with the multi-bed system showed a lower estimation of radiation uptake compared to that of the single-bed system when images were acquired for small lesions. However, phantom studies showed that there were no differences between the single- and multi-bed systems for volumes > 4 mm and that multi-bed systems can be applied in oncological and neurological studies. The multi-bed system may improve the efficacy of preclinical research by saving the time taken to acquire PET images.

## Data Availability

The data presented in this study are available on request from the corresponding author.
